# Role of KLHL3 and dietary K^+^ in regulating KS-WNK1 expression

**DOI:** 10.1152/ajprenal.00575.2020

**Published:** 2021-03-08

**Authors:** Mauricio Ostrosky-Frid, María Chávez-Canales, Jinwei Zhang, Olena Andrukhova, Eduardo R. Argaiz, Fernando Lerdo-de-Tejada, Adrian Murillo-de-Ozores, Andrea Sanchez-Navarro, Lorena Rojas-Vega, Norma A. Bobadilla, Norma Vazquez, María Castañeda-Bueno, Dario R. Alessi, Gerardo Gamba

**Affiliations:** ^1^Molecular Physiology Unit, Instituto de Investigaciones Biomédicas, Universidad Nacional Autónoma de México, Mexico City, Mexico; ^2^PECEM (MD/PhD), Facultad de Medicina, Universidad Nacional Autónoma de México, Mexico City, Mexico; ^3^Unidad de Investigación UNAM-INC, Instituto Nacional de Cardiología Ignacio Chávez and Instituto de Investigaciones Biomédicas, Universidad Nacional Autónoma de México, Mexico City, Mexico; ^4^Institute of Biomedical and Clinical Sciences, Medical School, College of Medicine and Health, University of Exeter, Hatherly Laboratories, Exeter, United Kingdom; ^5^MRC Protein Phosphorylation and Ubiquitylation Unit, College of Life Sciences, University of Dundee, Dundee, United Kingdom; ^6^Facultad de Medicina, Universidad Nacional Autónoma de México, Mexico City, Mexico; ^7^Department of Nephrology and Mineral Metabolism, Instituto Nacional de Ciencias Médicas y Nutrición Salvador Zubirán, Mexico City, Mexico

**Keywords:** distal convoluted tubule, hypertension, salt transport, STE20/SPS1-related proline-alanine-rich protein kinase, with no lysine kinase 4

## Abstract

The physiological role of the shorter isoform of with no lysine kinase (WNK)1 that is exclusively expressed in the kidney (KS-WNK1), with particular abundance in the distal convoluted tubule, remains elusive. KS-WNK1, despite lacking the kinase domain, is nevertheless capable of stimulating the NaCl cotransporter, apparently through activation of WNK4. It has recently been shown that a less severe form of familial hyperkalemic hypertension featuring only hyperkalemia is caused by missense mutations in the WNK1 acidic domain that preferentially affect cullin 3 (CUL3)-Kelch-like protein 3 (KLHL3) E3-induced degradation of KS-WNK1 rather than that of full-length WNK1. Here, we show that full-length WNK1 is indeed less impacted by the CUL3-KLHL3 E3 ligase complex compared with KS-WNK1. We demonstrated that the unique 30-amino acid NH_2_-terminal fragment of KS-WNK1 is essential for its activating effect on the NaCl cotransporter and recognition by KLHL3. We identified specific amino acid residues in this region critical for the functional effect of KS-WNK1 and KLHL3 sensitivity. To further explore this, we generated KLHL3-R528H knockin mice that mimic human mutations causing familial hyperkalemic hypertension. These mice revealed that the KLHL3 mutation specifically increased expression of KS-WNK1 in the kidney. We also observed that in wild-type mice, the expression of KS-WNK1 was only detectable after exposure to a low-K^+^ diet. These findings provide new insights into the regulation and function of KS-WNK1 by the CUL3-KLHL3 complex in the distal convoluted tubule and indicate that this pathway is regulated by dietary K^+^ levels.

**NEW & NOTEWORTHY** In this work, we demonstrated that the kidney-specific isoform of with no lysine kinase 1 (KS-WNK1) in the kidney is modulated by dietary K^+^ and activity of the ubiquitin ligase protein Kelch-like protein 3. We analyzed the role of different amino acid residues of KS-WNK1 in its activity against the NaCl cotransporter and sensitivity to Kelch-like protein 3.

## Graphical abstract

**Figure:**
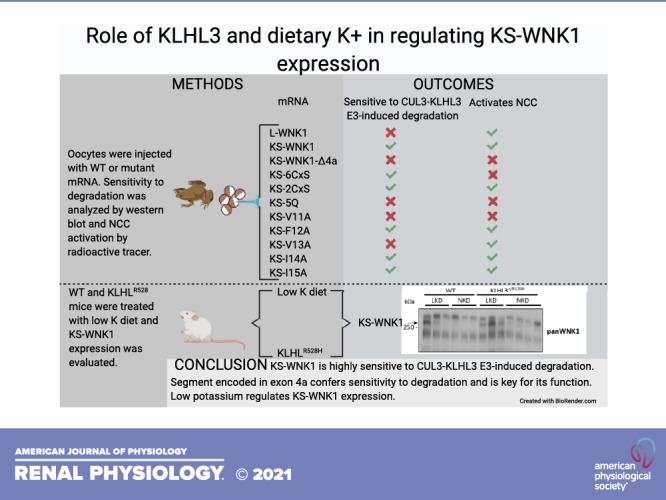

## INTRODUCTION

Familial hyperkalemic hypertension (FHHt) encompasses a spectrum of diseases that are mainly the consequence of overactivity of the renal thiazide-sensitive NaCl cotransporter (NCC) of the distal convoluted tubule (DCT) (1). NCC activity is modulated by with no lysine kinase (WNK)1 and WNK4, whose half-life is, in turn, regulated by the cullin–RING E3 ligase complex containing Kelch-like protein 3 (KLHL3) and cullin 3 (CUL3) proteins. The severity of FHHt depends on which one of these genes is affected and is defined by age of diagnosis, K^+^ levels, blood pressure levels, and percentage of affected individuals with hypertension before the age of 18 yr old. The more severe disease presentation is due to exon 9 deletion of CUL3, followed by dominant or recessive mutations in KLHL3 (2, 3). These mutations impair the ubiquitylation and degradation of WNK kinases in the DCT. Less severe is FHHt due to missense mutations in the acidic motif of WNK4, which constitutes the recognition site for KLHL3 (4, 5) and thus abrogate only WNK4 ubiquitylation (6). Finally, the mildest form of FHHt is due to intronic deletions in the *WNK1* gene that apparently result in ectopic expression of the full-length catalytic isoform of this kinase (known as L-WNK1) in the DCT (7). Louis-Dit-Picard et al. (8) have recently described, however, an even milder form of FHHt. In this work, humans and mice with heterozygous mutations in the acidic domain of WNK1 display an inherited phenotype with hyperkalemia, hyperchloremia, and metabolic acidosis, but without arterial hypertension, that is nevertheless accompanied by low renin expression levels, suggesting a mild volume expansion that is, however, not enough to produce hypertension.

The major product of the *WNK1* gene in the kidney is a shorter isoform known as kidney-specific WNK1 (KS-WNK1) (9–11). This isoform is transcribed from an alternative promoter located between exons 4 and 4a that lacks the kinase domain and contains a unique 30-amino acid residues sequence encoded by exon 4a. Despite lacking a kinase domain, recent evidence suggests that KS-WNK1 may function as an activator of NCC. First, in *Xenopus laevis* oocytes, we have shown that KS-WNK1 is able to induce phosphorylation and activation of NCC by interacting with other WNKs and reducing its sensitivity to Cl^−^. For instance, in the presence of KS-WNK1, WNK4 is active at higher levels of intracellular Cl^−^ concentration, increasing the activity of the intermediate kinase, STE20/SPS1-related proline-alanine-rich protein kinase (SPAK), toward NCC (12). Second, it has been observed that under conditions in which NCC activity is expected to be increased, like in response to low dietary K^+^, conglomerations of WNKs, known as WNK bodies, are formed in DCT cells, and their formation requires the presence of KS-WNK1 (13, 14), also supporting that KS-WNK1 is associated with activation of NCC. Third, Louis-Dit-Picard et al. (8) suggested that the FHHt phenotype observed in patients with mutations in the acidic domain of WNK1 is likely due to increased protein expression of KS-WNK1 in DCT cells because it was observed that the sensitivity of KS-WNK1 to the CUL3-KLHL3 E3 ligase complex is several times higher than that of L-WNK1 and, thus, mutations of the acidic motif in WNK1 seem to preferentially protect KS-WNK1 from ubiquitylation by the CUL3-KLHL3 E3 ligase complex.

Given that both KS-WNK1 and L-WNK1 contain the acidic motif involved in KLHL3 binding, the different sensitivity to CUL3-KLHL3 E3 ligase complex-mediated degradation is surprising. Our goal in the present study was to analyze and characterize the effect of the CUL3-KLHL3 E3 ligase complex on KS-WNK1 and to define the protein domains responsible for the difference in sensitivity. In addition, we began to explore the physiological stimuli that regulate KS-WNK1 protein expression levels by modulating its targeting to degradation by the CUL3-KLHL3 E3 complex.

## METHODS

### Generation of KLHL3^+/R528H^ Mice

*KLHL3*^+/R528H^ knockin mice were generated through homologous recombination strategies by TaconicArtemis (https://www.taconic.com/). For vector construction, mouse genomic fragments (obtained from the C57BL/6J RPCIB-731 BAC library) and selected features (such as desired point mutation, recombination sites, and selection markers, as provided in Supplemental Fig. S1; see https:/doi.org/10.6084/m9.figshare.13721791) were assembled into the targeting vector.

**Figure 1.**
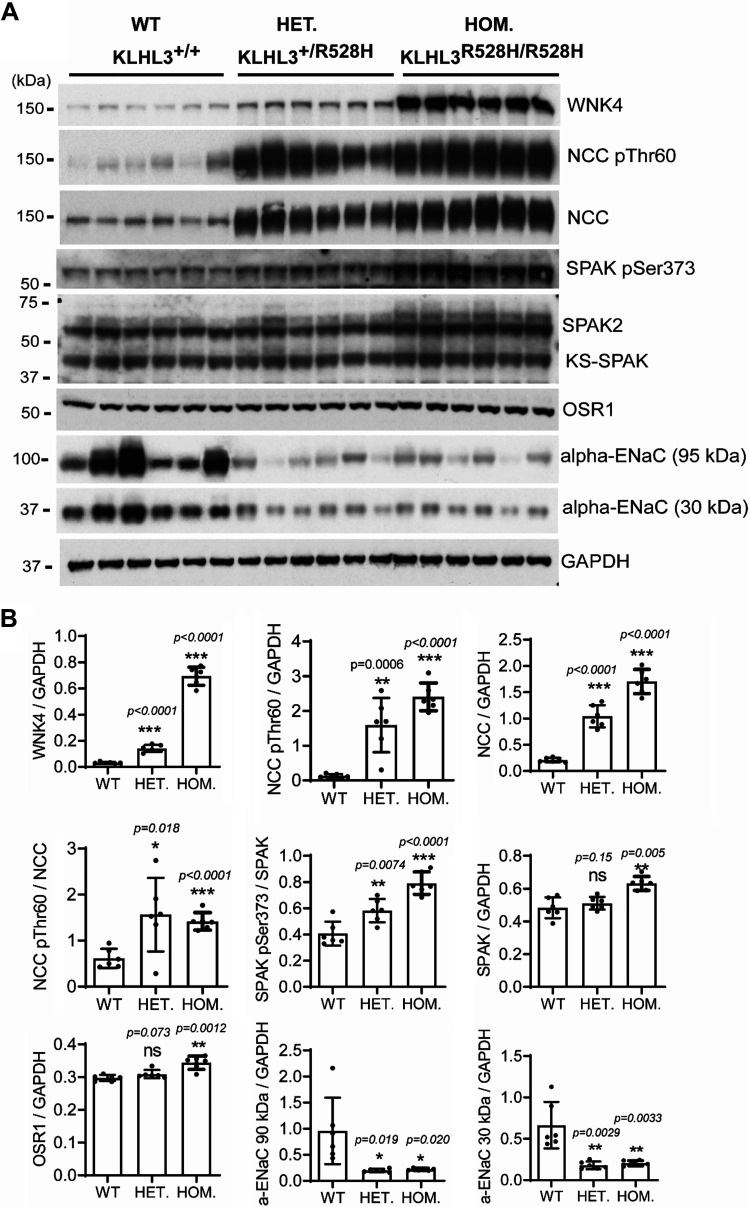
Kelch-like protein 3 (*KLHL3*)^+/R528H^ and *KLHL3^R528H^*^/R528H^ mice display the expected changes in the expression and phosphorylation levels of components of the with no lysine kinase 4 (WNK4)-STE20/SPS1-related proline-alanine-rich protein kinase (SPAK)/oxidative stress response-1 (OSR1)-NaCl cotransporter (NCC) pathway. *A*: total kidney extracts from wild-type (WT), *KLHL3*^+/R528H^ [heterozygrous (HET)] and *KLHL3^R528H^*^/R528H^ [homozygous (HOM)] mice were subjected to Western blot analysis with the indicated antibodies. Each sample was derived from a separate littermate animal. *B*: band intensities were quantified using ImageJ, and the results are presented relative to the expression of GAPDH. Increased expression of NCC and WNK4 as well as increased phosphorylation of SPAK (Ser^373^) and NCC (Thr^60^) were observed in *KLHL3*^+/R528H^ mice. Such differences were more dramatic in *KLHL3^R528H^*^/R528H^ mice, in which an increase in the expression of SPAK and OSR1 was also observed. ENaC, epithelial Na^+^ channel; ns, not significant.

The linearized vector was transfected into the TaconicArtemis C57BL/N Tac embryonic stem (ES) cell line. Homologous recombinant clones were isolated using positive (PuroR) and negative [thymidine kinase (TK)] selection. Specific ES clones were selected by Southern blot analysis of genomic DNA. Blastocysts were isolated from the uterus of pregnant BALB/c females at *day postcoitum 3.5* and injected with 10–15 targeted C57BL/6NTac ES cells. After recovery, eight injected blastocysts were transferred to each uterine horn of 2.5 days postcoitum, pseudopregnant Naval Medical Research Institute (NMRI) females. Chimerism was measured in chimeras (G_0_) by coat color contribution of ES cells to the BALB/c host (black/white). Highly chimeric mice were bred to C57BL/6-Tg(CAG-Flpe)2 Arte females for elimination of the puromycin resistance cassette. This produced mice that constitutively express mutated KLHL3 protein. The remaining FRT recombination site in these mice is located in a nonconserved region of the genome. Primers 6560_31 (5′- CACTGTGTTCTGCCTTTCAGG-3′) and 6560_32 (5′- CAGACCAAGACCAGAGAGAAGG-3′) were used to confirm the presence of the R528H mutation by PCR amplification and product sequencing.

For genotyping analysis, genomic DNA was extracted from tail biopsies and analyzed by PCR. *Primer 1* (5′- GATACCCACTGGCATTTGG-3′) and *primer 2* (5′- GGTAAGGGCAGCATTACTGG-3′) were used to detect wild-type and knockin alleles. The wild-type allele generates a 308-bp product, whereas the knockin allele generates a 383-bp product. The latter product is larger due to the presence of the FRT site and flanking region that remains in an intronic region following Flp-mediated excision of the puromycin selection cassette.

### Generation of KLHL3^R528H/R528H^/KS-WNK1^−/−^ Mice

*KS-WNK1*^−/−^ mice were a kind gift from Hadchouel (15) (INSERM Paris), and the generation and genotyping have been described in the supporting information of the cited article.

Mice were crossed with of *KLHL3*^+/R528H^ to produce the desired genotypes: wild type, *KLHL3^R528H^*^/R528H^, *KS-WNK1*^−/−^, and *KLHL3^R528H^*^/R528H^/*KS-WNK1*^−/−^. Male mice were used for experimental purposes.

### Low-K^+^ Diet Experiments

Wild-type and *KLHL3*^+/R528H^ mice were placed on normal (1% K^+^) or K^+^-deficient diets (0.0% K^+^) for 4 days. The OpenStandard diet with no added K^+^ (D16120202) was purchased from Research Diets and used as the K^+^-deficient diet. The normal-K^+^ diet was prepared by adding KCl. By the end of the 4-day period, mice were euthanized under isoflurane anesthesia, and kidney samples were collected.

### Western Blot Analysis of Mouse Kidney Proteins

Kidney lysates were prepared with lysis buffer containing 50 mM Tris-HCl (pH 7.5), 1 mM EGTA, 1 mM EDTA, 50 mM sodium fluoride, 5 mM sodium pyrophosphate, 1 mM sodium orthovanadate, 1% (w/v) Nonidet P-40, 0.27 M sucrose, 0.1% (v/v) 2-mercaptoethanol, and protease inhibitors (1 tablet per 50 mL). Lysates (20 µg) in SDS sample buffer were subjected to electrophoresis on polyacrylamide gels and transferred to nitrocellulose membranes. Membranes were incubated for 30 min with Tris-buffered saline-Tween 20 (TBS-T) containing 5% (w/v) skim milk. Membranes were then immunoblotted in 5% (w/v) skim milk in TBS-T with the indicated primary antibodies overnight at 4°C. Sheep antibodies were used at a concentration of 1–2 µg/mL. The incubation with phospho-specific sheep antibodies was performed with the addition of 10 µg/mL of the dephospho-peptide antigen used to raise the antibody. Blots were then washed six times with TBS-T and incubated for 1 h at room temperature with secondary horseradish peroxidase (HRP)-conjugated antibodies diluted 5,000-fold in 5% (w/v) skim milk in TBS-T. After the washing steps were repeated, the signal was detected with an enhanced chemiluminescence reagent. Immunoblots were developed using a film automatic processor (SRX-101, Konica Minolta Medical), and films were scanned with 600-dpi resolution on a scanner (PowerLook 1000, UMAX).

For the detection of KS-WNK1, kidney tissue was homogenized in lysis buffer containing 250 mM sucrose, 10 mM triethanolamine, 1× protease inhibitors (Roche), 50 mM sodium fluoride, 10 mM sodium pyrophosphate, and 1 mM sodium orthovanadate.

Protein samples were subjected to polyacrylamide gel electrophoresis and then transferred to PVDF membranes for 2 h at 10 mV. Membranes were blocked for 2 h in 10% nonfat dry milk dissolved in TBS-T solution (2 mM Tris-HCl, 150 mM NaCl, and 0.2% Tween 20, pH 7.5).

Membranes were incubated overnight with the indicated antibodies diluted in 5% nonfat dry milk in TBS-T, followed by incubation for 1 h at room temperature with HRP-conjugated secondary antibodies diluted in 5% nonfat dry milk in TBS-T. After the incubation, membranes were washed six times for 10 min with TBS-T. For signal detection by chemiluminescence, Luminata Forte Western HRP substrate (Merck Millipore) was used, and LI-COR equipment was used to perform the reading.

The following antibodies were used. Rabbit anti-WNK4 (1:4,000) was a gift from David Ellison (Oregon Health & Science University) (16). For WNK1 detection, we used commercially available rabbit anti-WNK1 antibody from Bethyl Laboratories (1:1,000, A301-515A, pan-WNK1). The following antibodies were raised in the sheep and affinity purified on the appropriate antigen by the Division of Signal Transduction Therapy Unit of the University of Dundee: WNK4 total antibody (S064B, second bleed, raised against residues 1,221−1,243 of human WNK4), NCC phospho-Thr^60^ antibody (S995B, residues 54–66 of human NCC phosphorylated at Thr^60^, RTFGYNpTIDVVPT), NCC total antibody (S965B, residues 906–925 of human NCC, CHTKRFEDMIAPFRLNDGFKD), SPAK NH_2_-terminal antibody (S668D, raised against residues 2−76 of mouse SPAK), oxidative stress response-1 (OSR1) mouse antibody (S149C, residues 389–408 of mouse OSR1, SAHLPQPAGQMPTQPAQVSL), SPAK/OSR1 (S motif) phospho-Ser^373^/Ser^325^ antibody [S670B, raised against residues 367–379 of human SPAK, RRVPGS(S)GHLHKT, which is highly similar to residues 319–331 of human OSR1, in which the sequence is RRVPGS(S)GRLHKT]. α-Epithelial Na^+^ channel (ENaC) antibody was kindly provided by Land (17); p44/42 MAPK (Erk1/2) antibody (3062) was purchased from Cell Signaling Technology. Secondary antibodies coupled to HRP used for immunoblot analysis were obtained from Pierce. All antibodies used and their corresponding validation studies are cited in Supplemental Table S1; see https://doi.org/10.6084/m9.figshare.13721797).

### Immunofluorescent Staining of Mouse Kidney Sections

Harvested mouse kidneys were immersion fixed in fresh 4% (w/v) formaldehyde-PBS (pH 6.9) for 16 h at 37°C, washed three times in PBS, and stored at 4°C until paraffin embedded. Sections (5 µm) were deparaffinized in Histoclear (National Diagnostics) and rehydrated in graded methanol steps. An antigen retrieval step was performed with R-Universal buffer in the 2100 antigen retriever for a single heat-pressure cycle (Aptum Biologics). Sections were permeabilized with 0.05% (v/v) Triton X-100-PBS for 20 min and blocked for 1 h at 37°C with 2% (v/v) donkey serum in 0.05% (v/v) Triton X-100-PBS. Primary antibodies were incubated overnight for 16 h at 4°C at the following concentrations diluted in 1% (v/v) donkey serum in 0.05% (v/v) Triton X-100-PBS: 2 µg/mL for total SPAK and phospho-SPAK S373 and anti-α-ENaC (Novus Biologicals, 1:500). Phospho-specific antibodies included the addition of 10 μg/mL of the nonphospho-peptide used to raise the antibody per 2 µg/mL of antibody used. Negative controls omitted the primary antibody and were processed in parallel. Slides were then washed for 20 min in 0.05% (v/v) Triton X-100-PBS and incubated in secondary antibody for 1 h at 37°C. Preabsorbed donkey IgG-conjugated Alexa Fluor 488, 633, and 647 secondary antibodies (Life Technologies/Abcam) were used at 1:200 diluted in 1% (v/v) donkey serum in 0.05% (v/v) Triton X-100-PBS for immunofluorescent labeling. Slides were washed as above, counterstained using Sytox orange nucleic acid stain (S11368, Life Technologies), mounted using Prolong gold antifade (P36930, Life Technologies), and shielded from light.

### Mutagenesis and Constructs

Rat NCC, human WNK4-Flag, human L-WNK1-Δ11-c-Myc, and human KS-WNK1-Δ11-c-Myc have been previously described (12, 18, 19). KS-WNK1-Δ4a, KS-WNK1-2CxS (KS-2CxS), KS-WNK1-6CxS (KS-6CxS), KS-WNK1-5Q (KS-5Q), KS-WNK1-V11A, KS-WNK1-F12A, KS-WNK1-V13A, KS-WNK1-I14A, and KS-WNK1-I15A were made from KS-WNK1-Δ11 using the QuikChange mutagenesis system (Stratagene). All modifications were confirmed by DNA sequencing. cRNA was made from linearized cDNA using the T7 RNA polymerase mMESSAGE kit (Ambion). The KLHL3-Flag clone was a gift from Richard P. Lifton (Rockefeller University). The open reading frame was subcloned into a pGEMHE vector.

### Functional Expression of NCC

Oocytes were extracted in clusters from adult female *X. laevis* frogs anesthetized by submerging them in 0.17% Tricaine. Oocytes were incubated with collagenase type 2 (3 mg/mL) eluted in Ca^2+^-free ND-96 (96 mM NaCl, 2 mM KCl, 1.0 mM MgCl_2_, and 5 mM HEPES, pH 7.4) for 1.5 h, washed three times with Ca^2+^-free ND-96, and incubated again with collagenase type 2 for 1.5 h. Oocytes were washed with ND-96 solution (96.0 mM NaCl, 2.0 mM KCl, 1.8 mM CaCl_2_, 1.0 mM MgCl_2_, and 5.0 mM HEPES, pH 7.4) three times and incubated overnight at 16°C.

Oocytes were injected with 20 ng of each of the indicated cRNAs and then incubated at 16°C for 48 h in ND-96 before protein extraction for Western blot analysis or 72 h before transport experiments. MG132 (100 µM) was added to the media 16 h before protein extraction in the described cases.

### Consent for the Performance of Animal Experiments

The use of *X. laevis* oocytes as well as wild-type and transgenic mice were approved by Institutional Animal Care and Use Committee of the Instituto Nacional de Ciencias Medicas y Nutricion Salvador Zubiran and in accordance with regulations set by the Universities of Cambridge and Dundee and the United Kingdom Home Office. Only male mice at 12–16 wk old were used.

### Western Blot Analysis of *X. laevis* Oocyte Proteins

Twenty oocytes per experimental group were collected, and samples were extracted using 5 µL/oocyte of lysis buffer containing 50 mM Tris-HCl (pH 7.5), 1 mM EGTA, 1 mM EDTA, 50 mM sodium fluoride, 10 mM sodium pyrophosphate, 1 mM sodium orthovanadate, 1% (w/v) Nonidet P-40, 0.27 M sucrose, and protease inhibitors (Complete tablets, Roche).

Western blots were performed as described above. The antibody concentrations used were anti-Flag 1:5,000 (Sigma), anti-Myc 1:1,000 (Sigma), and anti-actin 1:2,500 (Santa Cruz Biotechnology). Densitometric analysis was performed using ImageStudioLite software.

### Transport Assays

NCC activity was evaluated using the radioactive tracer ^22^Na^+^ (Perkin Elmer Life Sciences). Oocytes were injected as previously described; 72 h later, 15 oocytes per group were incubated at room temperature for 30 min in Cl^−^-free ND-96 medium [containing (in mM) 96 sodium isethionate, 2 potassium gluconate, 1.8 calcium gluconate, 1 magnesium gluconate, and 5 HEPES, pH 7.4] containing 1 mM ouabain, 100 µM amiloride, and 100 µM bumetanide in the presence or absence of 100 µM trichlormethiazide. Oocytes were transferred to K^+^-free uptake medium [containing (in mM) 40 NaCl, 56 NMDG-Cl, 1.8 CaCl_2_, 1 MgCl_2_, and 5 HEPES, pH 7.4] with ouabain, amiloride, and bumetanide with or without trichlormethiazide and with 0.5 μCi of ^22^Na^+^ for 60 min at 32°C. Oocytes were washed five times in an ice-cold radioactive-free medium and placed in individual tubes with 1% SDS. After lysis scintillation counting, liquid (ecolume, MP Biomedicals) was added.

### Statistical Analysis

In experiments with *n* ≥ 3, statistical significance was calculated with one-way ANOVA with multiple comparisons using GraphPad Prism 8.4.3. Significance was defined as *P* ≤ 0.05. Results are presented as means ± SEM.

## RESULTS

### Generation and Characterization of a New Strain of KLHL3 Knockin Mice

KLHL3 knockin mice carrying the FHHt mutation R528H that prevents binding to WNK kinases (20, 21) were generated by TaconicArtemis (http://www.taconic.com/wmspage.cfm?parm1=1453).

**Figure 2.**
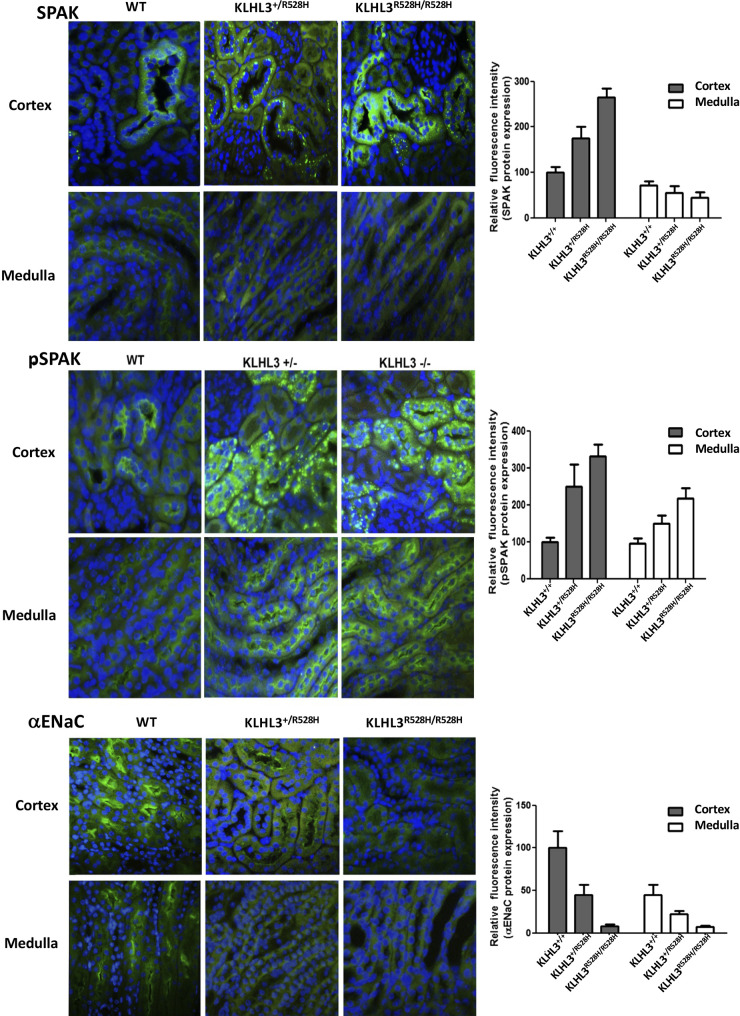
Immunofluorescent staining of kidney sections was performed. The primary antibodies used are indicated. Representative images from cortical and medullary regions are shown. Fluorescence intensity was quantified using ImageJ, and the results are presented in the corresponding bar graphs. ENaC, epithelial Na^+^ channel; KLHL3, Kelch-like protein 3; pSPAK, phospho-SPAK; SPAK, STE20/SPS1-related proline-alanine-rich protein kinase; WNK1, with no lysine kinase 1.

Phenotypic characterization under basal conditions showed that the mice displayed the expected FHHt-like phenotype with hyperkalemia, metabolic acidosis, and hyperchloremia (Table 1). The abundance and phosphorylation of relevant renal transporters and regulatory proteins were also studied. As previously reported for a *KLHL3*^+/R528H^ strain generated by Susa and collaborators (22), higher WNK4 and NCC expression levels as well as higher NCC (Thr^60^) and SPAK (Ser^373^) phosphorylation levels were observed in *KLHL3*^+/R528H^ mice compared with wild-type mice. These differences were more dramatic in *KLHL3^R528H^*^/R528H^ mice (Fig. 1). In homozygotes, SPAK and OSR1 expression levels were also higher than in wild-type mice. Additionally, in contrast to what was reported by Susa et al., a clear decrease in the abundance of the full-length and cleaved forms of α-ENaC was observed in both *KLHL3*^+/R528H^ and *KLHL3^R528H^*^/R528H^ mice made for this study. This finding was corroborated by immunofluorescent staining of kidney sections (Fig. 2). Finally, in sections stained with SPAK and phospho-SPAK (Ser^373^) antibodies, a punctate signal was observed in the cytoplasm of some cortical tubular cells of *KLHL3*^+/R528H^ and *KLHL3^R528H^*^/R528H^ mice that contrasted with the apical signal observed in wild-type mice. In the medullary portion, an apical expression pattern was observed in some tubules of wild-type and mutant mice, and this signal intensity was higher in mutant mice.

**Table 1.** Serum electrolytes of wild-type, KLHL3^+/R528H^, and KLHL3^R528H/R528H^ mice

	KLHL3^+/+^	KLHL3^+/R528H^	KLHL3^R528H^^/R528H^
Na^+^, mM	146.6 ± 1.06 (*n* = 17)	147.3 ± 0.45 (*n* = 28)	148.5 ± 0.77 (*n* = 16)
K^+^, mM	4.63 ± 0.09 (*n* = 18)	5.40 ± 0.1 (*n* = 28)*	5.56 ± 0.13 (*n* = 16)*
Cl^−^, mM	115.3 ± 1.46 (*n* = 17)	123.8 ± 1.94 (*n* = 24)*	123.3 ± 2.03 (*n* = 14)*
Ca^2+^, mM	2.21 ± 0.05 (*n* = 4)	2.28 ± 0.05 (*n* = 5)	2.36 ± 0.09 (*n* = 5)
pH	7.32 ± 0.03 (*n* = 6)	ND	7.18 ± 0.03 (*n* = 3)*

Data are means ± SE; *n* represents the number of mice included in the analyses. KLHL3, Kelch-like protein 3.

*P < 0.05 versus wild-type mice.

### KS-WNK1 Is Highly Sensitive to CUL3-KLHL3 E3 Ligase Complex-Mediated Degradation in Vivo

To analyze the effect of the KLHL3-R528H mutation on WNK1 expression, we used an antibody directed against a COOH-terminal epitope of the protein that can recognize both L-WNK1 and KS-WNK1 (pan-WNK1 antibody). Given that several bands were observed in the blots performed with this antibody, in order to identify the band corresponding to KS-WNK1, we included in these experiments lysates from KS-WNK1 knockout mice [*KS-WNK1*^−/−^, a kind gift of Hadchouel (15), INSERM Paris] and *KLHL3^R528H^*^/R528H^; *KS-WNK1*^−/−^ double mutants.

**Figure 3.**
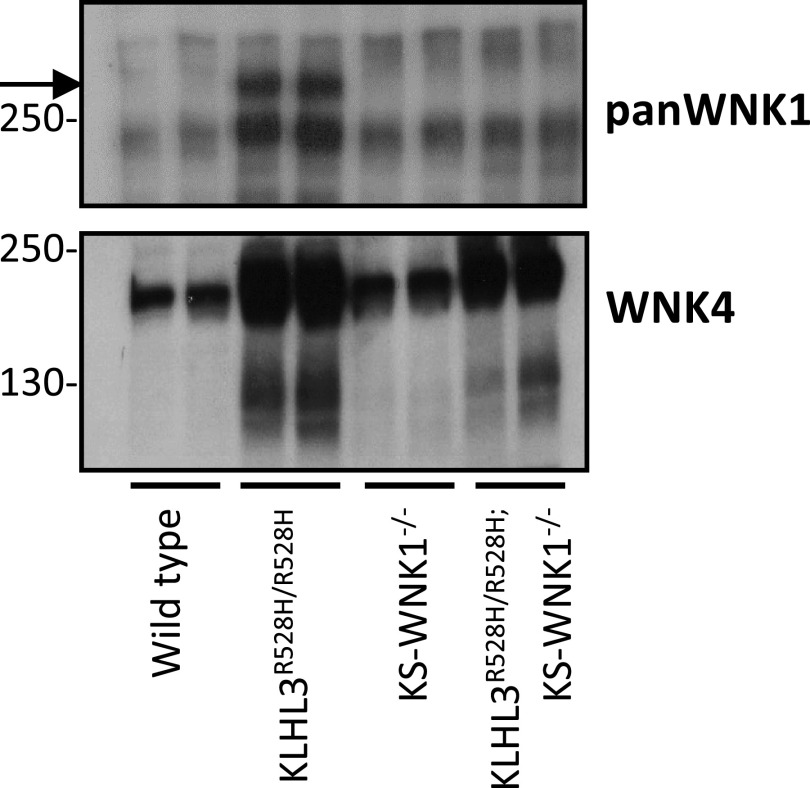
Kidney-specific with no lysine kinase (WNK)1 (KS-WNK1) protein levels are undetectable in kidney tissue of wild-type mice but are high in mice in which cullin 3-Kelch-like protein 3 (KLHL3) E3-mediated degradation is prevented. Total kidney lysates from *KLHL3^R528H^*^/R528H^ mice, *KS-WNK1*^−/−^ mice, and double mutants (*KLHL3^R528H^*^/R528H^; *KS-WNK1*^−/−^ mice) were analyzed by Western blot to assess the expression of WNK1 isoforms (as measured by the pan-WNK1 antibody). The robust band observed in *KLHL3^R528H^*^/R528H^ mice (arrow) that was absent in wild-type mice corresponds to KS-WNK1, as corroborated by its absence in the double mutants. The WNK4 blot is presented at the *bottom*. The expected increase in WNK4 expression was observed in *KLHL3^R528H^*^/R528H^ samples and in *KLHL3^R528H^*^/R528H^; *KS-WNK1*^−/−^ mice.

Interestingly, we observed no band at the expected size for KS-WNK1 in wild-type mice, but a robust band of this size was observed in *KLHL3^R528H^*^/R528H^ mice (Fig. 3). Its absence in double-mutant mice confirmed that indeed this band corresponds to KS-WNK1. These observations suggest that the KS-WNK1 protein expression level is very low (undetectable by Western blot) in kidneys from wild-type mice under basal conditions, which is striking given the high KS-WNK1 mRNA levels that have been reported for renal tissue (11). This observation supports, as suggested by Luis-Dit-Picard et al. (8), that KS-WNK1 is highly sensitive to CUL3-KLHL3 E3-mediated degradation, given the large upregulation observed in *KLHL3^R528H^*^/R528H^ mice in which WNK-KLHL3 binding is impaired.

Finally, a band of higher molecular weight that may correspond to L-WNK1 was observed with the pan-WNK1 antibody, whose intensity was similar in all genotypes. We cannot rule out, however, that this may be a nonspecific band. WNK4 expression in the kidneys from wild-type and *KS-WNK1*^−/−^ mice was similar, whereas, as expected, it was increased in *KLHL3^R528H^*^/R528H^ mice and double-mutant mice.

### Knockout of KS-WNK1 Does Not Prevent the FHHt Phenotype of KLHL3^R528H/R528H^ Mice

Given the striking KS-WNK1 protein upregulation that we observed in *KLHL3^R528H^*^/R528H^ mice, we decided to evaluate whether this upregulation is implicated in the pathogenesis of FHHt. Thus, we studied the phenotype of *KLHL3^R528H^*^/R528H^; *KS-WNK1*^−/−^ mice and compared it with the phenotype of *KLHL3^R528H^*^/R528H^ mice. We observed that double-knockout mice have an FHHt phenotype with serum K^+^, Cl^−^, HCO_3_^−^, and pH levels similar to those observed in *KLHL3^R528H^*^/R528H^ mice (Table 2). Thus, KS-WNK1 upregulation does not seem to play a central role in the pathogenesis of FHHt. In further experiments, administration of diets with altered content of K^+^ and Na^+^ may help to uncover phenotypic differences. However, this was out of the scope of this work.

**Table 2.** Serum electrolytes of wild-type, KLHL3^R528H/R528H^, KS-WNK1^−/−^, and double-mutant mice

	KLHL3^+/+^; KS-WNK1^+/+^	KLHL3^R528H^^/R528H^; KS-WNK1^+/+^	KLHL3^+/+^; KS-WNK1^−/−^	KLHL3^R528H^^/R528H^; KS-WNK1^−/−^
Na^+^, mM	151 ± 0.56 (*n* = 6)	152 ± 0.56 (*n* = 6)	150 ± 0.61 (*n* = 6)	153 ± 0.58 (*n* = 6)
K^+^, mM	4.5 ± 0.16 (*n* = 6)	5.1 ± 0.10 (*n* = 6)*	4.7 ± 0.12 (*n* = 6)	5.0 ± 0.12 (*n* = 6)*
Cl^−^, mM	118 ± 0.61 (*n* = 6)	123 ± 0.22 (*n* = 6)*	118 ± 0.53 (*n* = 6)	122 ± 0.40 (*n* = 6)*
Ca^2+^, mM	4.2 ± 0.3 (*n* = 6)	4.5 ± 0.06 (*n* = 6)	4.2 ± 0.12 (*n* = 6)	4.3 ± 0.04 (*n* = 6)
pH	7.3 ± 0.03 (*n* = 6)	7.2 ± 0.01 (*n* = 6)	7.3 ± 0.02 (*n* = 6)	7.2 ± 0.01 (*n* = 6)
HCO_3_^−^, mM	20 ± 0.76 (*n* = 6)	14 ± 0.42 (*n* = 6)*	17 ± 0.36 (*n* = 6)	15 ± 0.52 (*n* = 6)*

Data are means ± SE; *n* represents the number of mice included in the analyses. KLHL3, Kelch-like protein 3; WNK1, with no lysine kinase 1.

*P < 0.05 versus wild-type mice.

### KS-WNK1 and L-WNK1 Exhibit Different Sensitivity to CUL3-KLHL3 E3-Mediated Degradation

**Figure 4.**
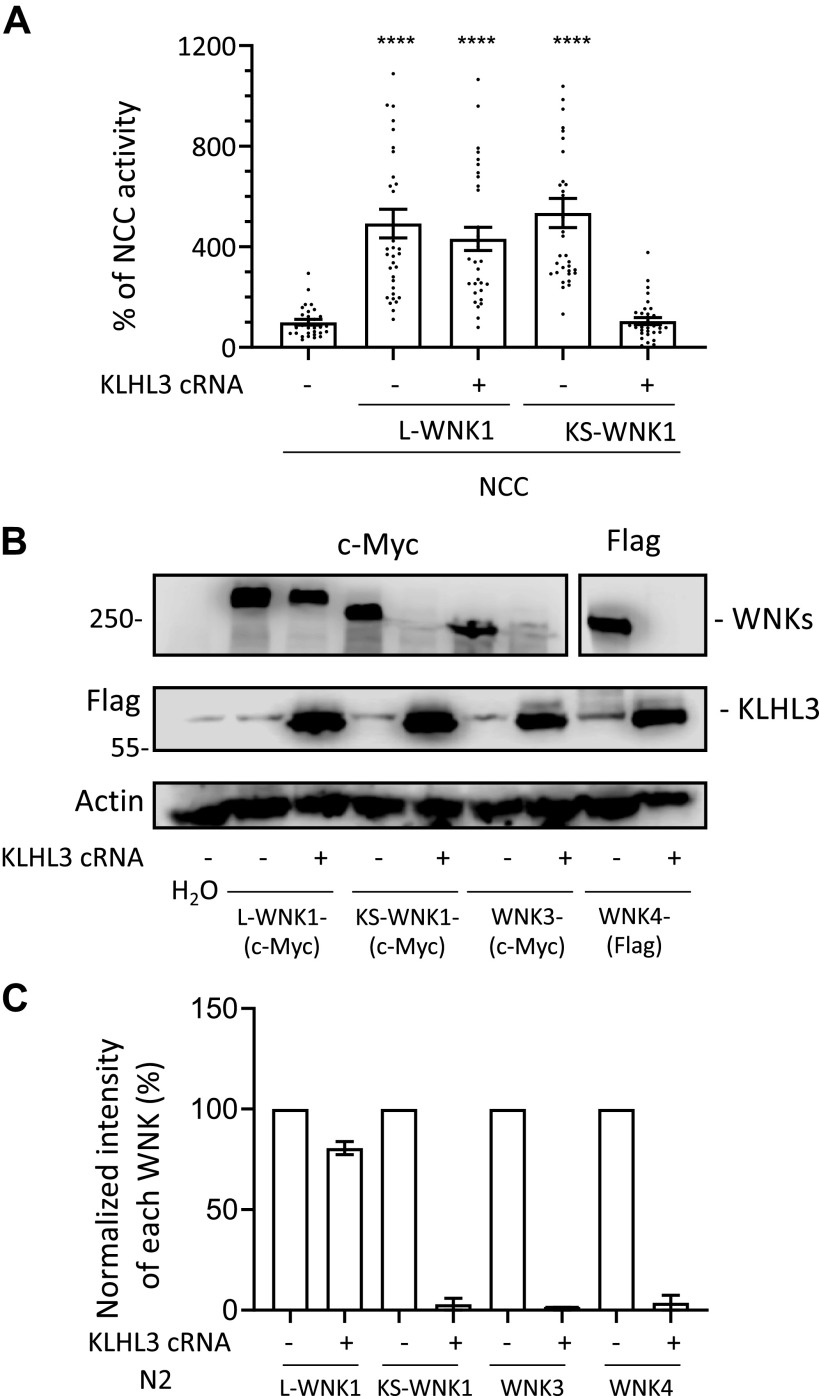
Kidney-specific with no lysine kinase (WNK)1 (KS-WNK1), WNK3, and WNK4, but not full-length WNK1 (L-WNK1), are degraded when coexpressed with Kelch-like protein 3 (KLHL3) in oocytes. *A*: thiazide-sensitive ^22^Na^+^ uptake was assessed in *Xenopus laevis* oocytes injected with the indicated cRNAs. Uptake values observed in the control group (NCC only) were set to 100%, and the other groups were normalized accordingly. While both L-WNK1 and KS-WNK1 increased Na^+^ uptake, KLHL3 coexpression prevented NaCl cotransporter (NCC) activation by KS-WNK1 but not by L-WNK1. Dots represent uptake values for individual oocytes. At least three independent experiments were performed with >10 oocytes per group (****P < 0.0001 vs. NCC; three points outside graphic limits). *B*: representative Western blots showing the effect of KLHL3 coexpression on L-WNK1, KS-WNK1, WNK3, and WNK4 levels. Oocytes were injected with cRNAs encoding for c-Myc-tagged L-WNK1, KS-WNK1, or WNK3 or Flag-tagged WNK4 with or without Flag-tagged KLHL3. All kinases except L-WNK1 were degraded in the presence of KLHL3. *C*: densitometric analysis of the Western blots presented in *B*. Two independent experiments were performed with similar results. c-Myc-tagged L-WNK1, KS-WNK1, or WNK3 or Flag-tagged WNK4 were normalized to 100% and compared with those observed in groups expressing KLHL3.

Our preliminary data, published in Louis-Dit-Picard et al. (8), showed that KS-WNK1 is heterologously expressed in *X. laevis* oocytes and in human embryonic kidney (HEK)-293 cells is readily degraded when coexpressed with KLHL3. In contrast, L-WNK1 is resistant to such degradation. To further explore this phenomenon, we began by analyzing the effect of KLHL3 coexpression on KS-WNK1 and L-WNK1-mediated activation of NCC. We microinjected *X. laevis* oocytes with NCC cRNA in the absence or presence of L-WNK1 or KS-WNK1 cRNA with or without KLHL3 cRNA. Three days later, thiazide-sensitive tracer Na^+^ uptake was assessed. As we have previously shown (12), both KS-WNK1 and L-WNK1 were able to increase the activity of NCC, despite the fact that KS-WNK1 has no kinase domain (Fig. 4*A*). Our previous work supported that the effect of KS-WNK1 on NCC is likely due to an interaction of KS-WNK1 with an endogenous WNK kinase, since the presence of KS-WNK1 increased the phosphorylation of SPAK and NCC, and the effect was prevented by the specific WNK inhibitor WNK463 (12). Consistent with our preliminary observations (8), the effect of KS-WNK1 on NCC was completely prevented by coinjection with KLHL3 cRNA, whereas the effect of L-WNK1 on NCC was not.

We then analyzed the effect of KLHL3 expression on L-WNK1, KS-WNK1, WNK3, and WNK4 abundances. This effect has been previously described. However, no study has compared the effect of KLHL3 on all these WNK isoforms in parallel (4, 5, 21). Figure 4*B* shows a representative image of such analysis. Oocytes were injected with each WNK cRNA alone or coinjected with KLHL3 cRNA. L-WNK1, KS-WNK1, and WNK3 expression was assessed with anti-c-myc antibodies and WNK4 and KLHL3 with anti-Flag antibodies. Consistent with the results shown in Fig. 4*A*, the presence of KLHL3 had little to no effect on the L-WNK1 expression level, whereas the expression of KS-WNK1 in the presence of KLHL3 was completely abrogated. In addition, we observed that WNK3 and WNK4 exhibited high sensitivity to CUL3-KLHL3 E3-mediated degradation. Thus, according to the densitometric analysis (Fig. 4*C*), it appears that at least in *X. laevis* oocytes the sensitivity of L-WNK1 to the effect of the CUL3-KLHL3 E3 complex was significantly lower than that observed for the other WNKs. A similar observation has also been reported in HEK-293 cells by Louis-Dit-Picard et al. (8).

### The Unique KS-WNK1 Segment Encoded by Exon 4a Is Involved in the Sensitivity to the CUL3-KLHL3 E3 Ligase Complex

L-WNK1 and KS-WNK1 have different NH_2_-terminal portions but are identical from the beginning of the segment encoded by exon 5 until the end of the protein. Thus, they both contain the acidic motif (EPEEPEADQHQ) that mediates interactions with KLHL3 (Supplemental Fig. S2; see https://doi.org/10.6084/m9.figshare.13721794).

**Figure 5.**
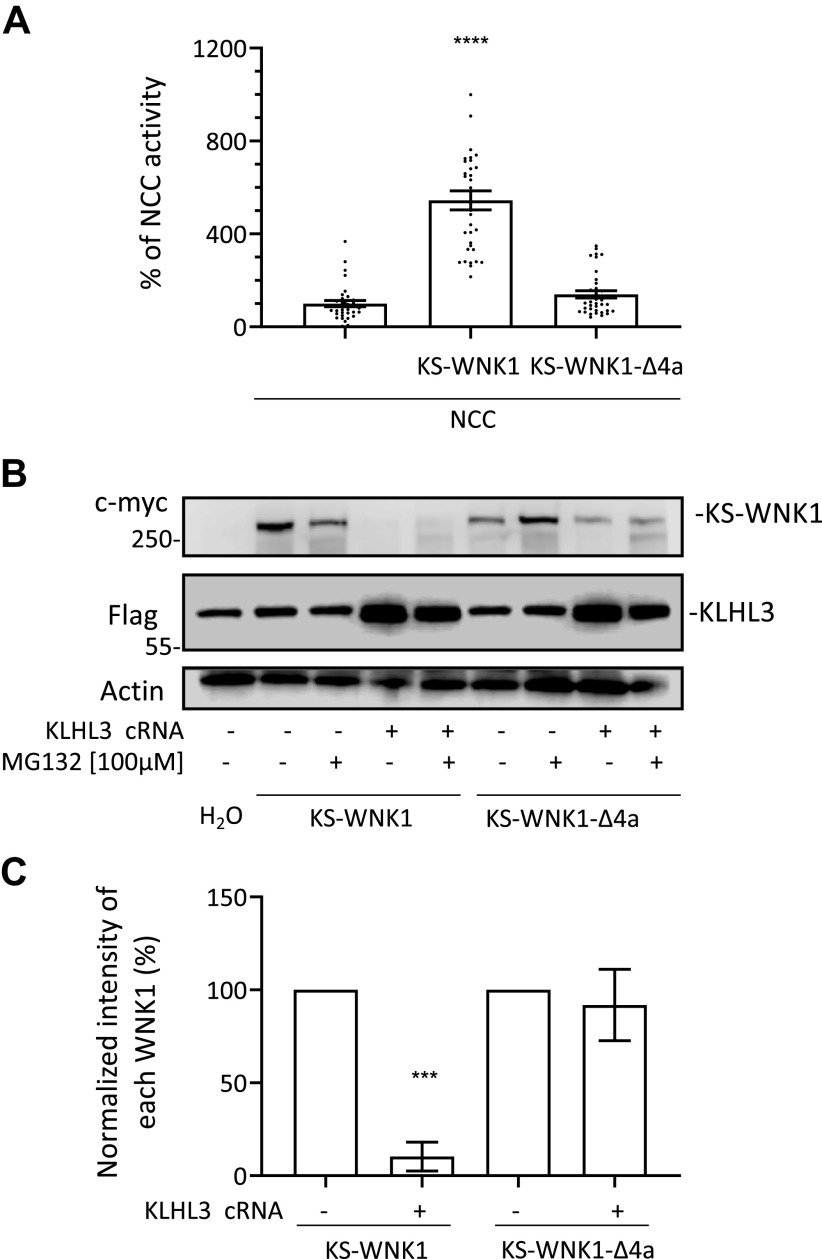
The segment encoded by exon 4a in kidney-specific with no lysine kinase 1 (KS-WNK1) is needed to activate NaCl cotransporter (NCC) and to be targeted for degradation by cullin-3 (CUL3)–Kelch-like protein 3 (KLHL3) E3. *A*: thiazide-sensitive Na^+^ uptake of NCC cRNA-injected oocytes was set to 100%, and uptake values of additional groups were normalized accordingly. KS-WNK1 coexpression increased NCC activity, but the KS-WNK1-Δ4a mutant failed to activate (*n* = 3 transport assays, ****P < 0.0001 vs. NCC; one point outside graphic limits). *B*: representative Western blots showing KS-WNK1 and KS-WNK1-Δ4a expression in the absence or presence of KLHL3. CUL3-KLHL3 E3-induced degradation of KS-WNK1 is observed regardless of proteosome inhibition, whereas KS-WNK1-Δ4a is expressed but resistant to CUL3-KLHL3 E3-induced degradation. *C*: densitometric analysis of the Western blots presented in *B*. Results from four different experiments were included. Expression of KS-WNK1 or KS-WNK1-Δ4a in the absence of KLHL3 were arbitrarily set to 100% and compared with expression levels observed in the presence of KLHL3 (*n* = 4 Western blots, ***P > 0.001 vs. control without KLHL3).

The first 30-amino acid residues of KS-WNK1 are unique to this isoform because they are encoded by exon 4a, which is not included in the L-WNK1 transcript. Exon 4a is highly conserved across evolution since its amino acid sequence is almost identical from coelacanths to humans (13), suggesting that it plays a key role in species that have evolved a renal tubule. This segment has been shown to be key for the formation of KS-WNK1-dependent WNK bodies (13). Thus, to evaluate its effect on KS-WNK1 activity and CUL3-KLHL3 E3-mediated degradation, we generated the KS-WNK1-Δ4a clone that lacks this fragment. We assessed its ability to activate NCC and its sensitivity to degradation promoted by CUL3-KLHL3 E3. As previously described (12), the absence of the 4a fragment prevents the positive effect of KS-WNK1 on NCC activity (Fig. 5*A*). Interestingly, this modification prevented KS-WNK1 functionality and also prevented its degradation, as demonstrated by the representative blot shown in Fig. 5*B* and the densitometric analysis shown in Fig. 5*C*. We observed a mild protective effect of the proteasome inhibitor MG132 against CUL3-KLHL3-RING-induced degradation of KS-WNK1, suggesting that other pathways may also be involved in degradation (Fig. 5*B*).

### The Cysteines in Region 4a Are Important for KS-WNK1 Function but Not for Its CUL3-KLHL3 E3-Mediated Degradation

**Figure 6.**
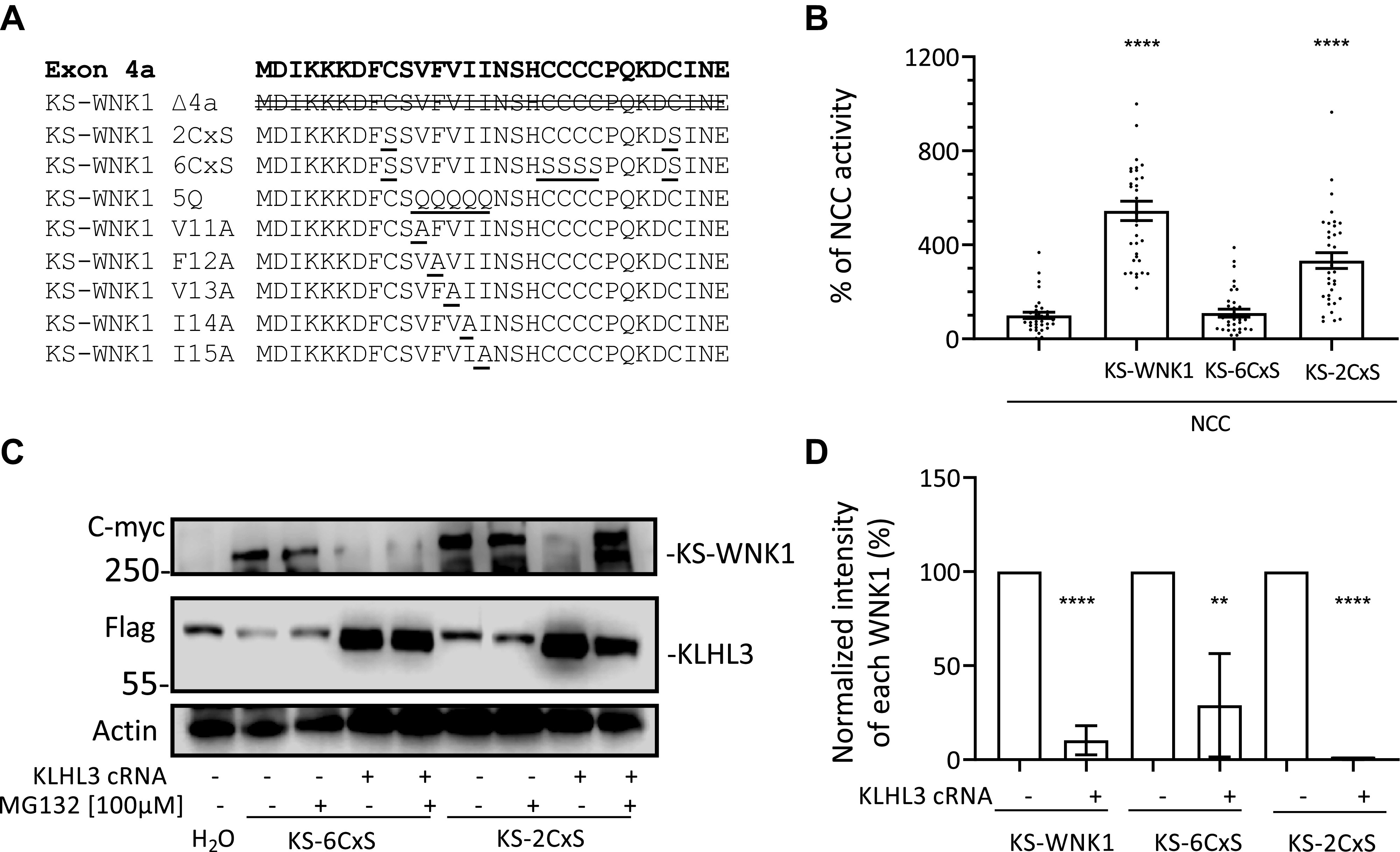
Mutation of the six conserved cysteines encoded in exon 4a impairs the ability of kidney-specific with no lysine kinase 1 (KS-WNK1) to activate NaCl cotransporter (NCC) but does not prevent cullin-3 (CUL3)-Kelch-like protein 3 (KLHL3) E3-induced degradation. *A*: amino acid sequence encoded by exon 4a. Different KS-WNK1 mutants were generated for this work with variations in the sequence of this region. The modifications introduced in each mutant are indicated. *B*: NCC was coexpressed in oocytes with KS-WNK1, KS-6CxS (in which all six cysteines were mutated to serine), or KS-2CxS (in which the two peripheral cysteines were mutated to serine). Thiazide-sensitive Na^+^ uptake of NCC-expressing oocytes was set to 100% and compared with all other groups, which were normalized accordingly. KS-WNK1-6CxS did not activate NCC, whereas KS-WNK1-2CxS did activate NCC, albeit at a lower level than wild-type KS-WNK1 (*n* = 3 transport assays, ****P < 0.0001 vs. NCC; 3 points outside graphic limits). *C*: representative Western blots showing the expression of KS-WNK1-6CxS and KS-WNK1-2CxS. Both mutant proteins are targeted for degradation by CUL3-KLHL3 E3, whereas treatment with MG132 could prevent KS-WNK1-2CxS degradation. *D*: compiled results of densitometric analysis from at least two different Western blot experiments like that presented in (*B*). Expression levels of KS-WNK1, KS-WNK1-6CxS, and KS-WNK1-2CxS in the absence of KLHL3 were normalized to 100% and compared with groups expressing KLHL3 (*n* = 2−4 Western blots, **P < 0.01 and ****P < 0.0001 vs. control without KLHL3).

Boyd-Shiwarski et al. (13) have previously analyzed the degree of conservation of individual residues within the 4a segment and have identified a cluster of conserved cysteines and a cluster of conserved hydrophobic residues (Fig. 6*A*). Mutagenic analysis led them to conclude that these clusters, which they termed the “cysteine-rich hydrophobic motif,” are key for the formation of KS-WNK1-dependent WNK bodies. Thus, we decided to evaluate their role on KS-WNK1-dependent NCC activation and sensitivity to CUL3-KLHL3 E3-induced degradation.

We first decided to analyze the role of conserved cysteines. For this purpose, we generated a clone with the six conserved cysteines mutated to serine (KS-6CxS mutant) and a clone in which only the outer two cysteines were mutated to serine (KS-2CxS mutant; Fig. 6*A*). According to the data by Boyd-Shiwarski et al. (13), the KS-6CxS mutation prevents the formation of WNK bodies, but the KS-2CxS mutation only partially does so.

We injected oocytes with NCC and wild-type KS-WNK1 or mutants KS-6CxS or KS-2CxS and treated them with or without MG132 to evaluate their function and degradation. We observed that the ability of the KS-6CxS mutant to activate NCC was completely abrogated, whereas that of the KS-2CxS mutant was only partially impaired (Fig. 6*B*). Regarding CUL3-KLHL3 E3-mediated degradation, we observed that both mutants were degraded in the presence of KLHL3, suggesting that, although the whole exon 4a seems to be important in conferring sensitivity to CUL3-KLHL3 E3-mediated degradation, the cysteine residues are not involved (Fig. 6, *C* and *D*).

### The Cluster of Hydrophobic Residues in the 4a Segment of KS-WNK1 Are Key for NCC Activation and Confers KS-WNK1 Sensitivity to CUL3-KLHL3 E3-Mediated Degradation

**Figure 7.**
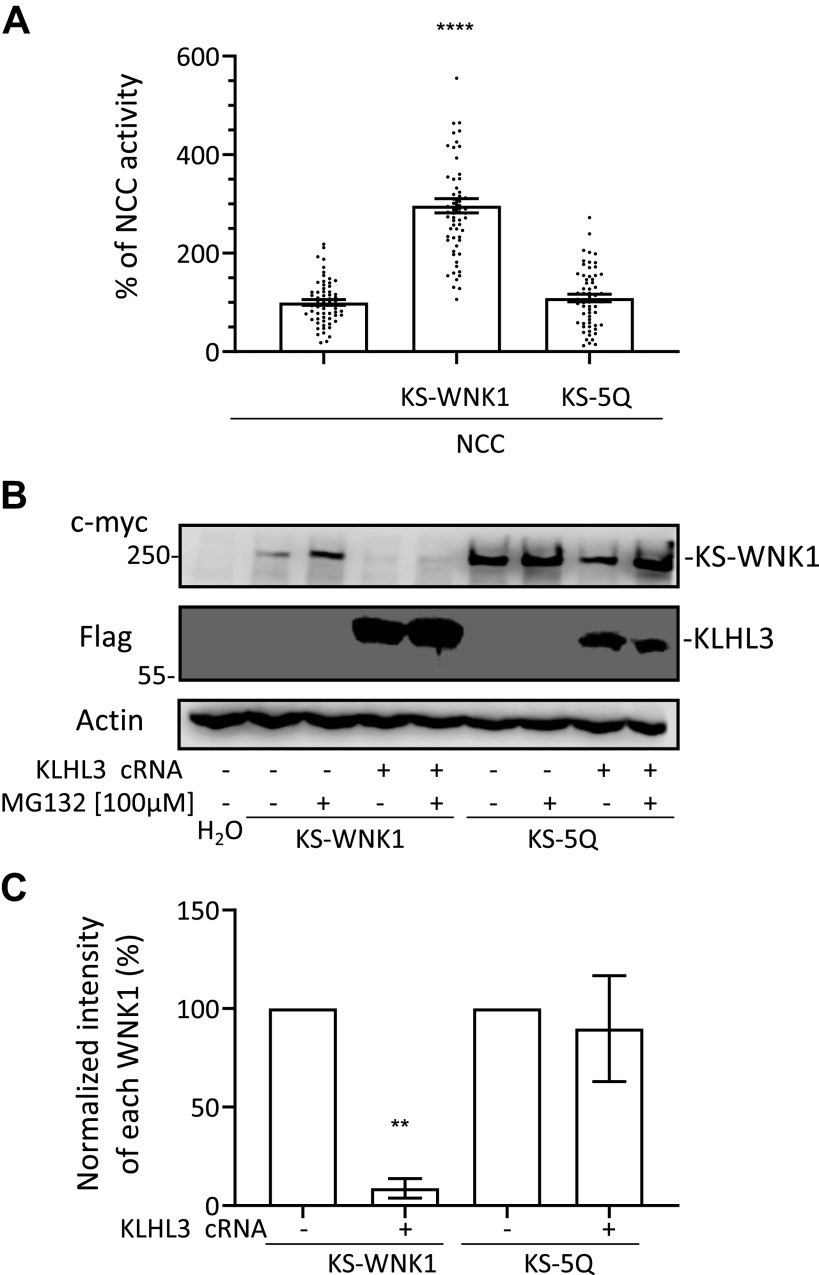
Mutation of the five conserved hydrophobic residues encoded in exon 4a impairs the ability of kidney-specific with no lysine kinase 1 (KS-WNK1) to activate NaCl cotransporter (NCC) and prevent cullin-3-Kelch-like protein 3 (KLHL3) E3-induced degradation. *A*: NCC was coexpressed in oocytes with KS-WNK1 or KS-5Q (in which all five hydrophobic residues were mutated to glutamine), and thiazide-sensitive Na^+^ uptake was assessed. Uptake levels observed for NCC-expressing oocytes were normalized to 100% and compared with those observed for groups expressing KS-WNK1 and KS-5Q. The strong activation of NCC induced by KS-WNK1 was not observed in the KS-5Q mutant (*n* = 5 transport assays, ****P < 0.0001 vs. NCC; 2 points outside graphic limits). *B*: representative Western blots showing the expression of KS-WNK1 and KS-5Q. KS-WNK1 was degraded in the presence of KLHL3, whereas KS-5Q was not degraded. Treatment with MG132 increased KS-5Q expression, probably by impairing degradation even more. *C*: compiled results of densitometric analysis from three different experiments like that presented in *B*. Expression levels of KS-WNK1 and KS-5Q in the absence of KLHL3 were normalized to 100% and compared with those observed in groups expressing KLHL3 (*n* = 3 Western blots, **P < 0.01 vs. control without KLHL3).

We next analyzed the role of the cluster of hydrophobic residues on KS-WNK1 ability to activate NCC and on its sensitivity to CUL3-KLHL3 E3-mediated degradation. This cluster includes the five hydrophobic amino acid residues between the positions 11 and 15 (valine, phenylalanine, valine, isoleucine, and isoleucine; Fig. 6*A*). We generated a clone in which these five residues were mutated to glutamine (KS-5Q mutant). We decided to mutate these residues to glutamine given that Boyd-Shiwarski et al. (13) used this strategy to substitute the hydrophobic residues for hydrophilic ones and showed that these mutations prevented WNK body formation. Thus, we evaluated if the same mutations can also affect kidney-specific function and KLHL3-induced degradation. We observed that the ability of this mutant to activate NCC was completely impaired (Fig. 7*A*). Interestingly, however, we observed that this mutant was insensitive to CUL3-KLHL3 E3-induced degradation (Fig. 7, *B* and *C*).

### Valine 11 and Valine 13 of KS-WNK1 Are Relevant for Its CUL3-KLHL3 E3-Mediated Degradation

**Figure 8.**
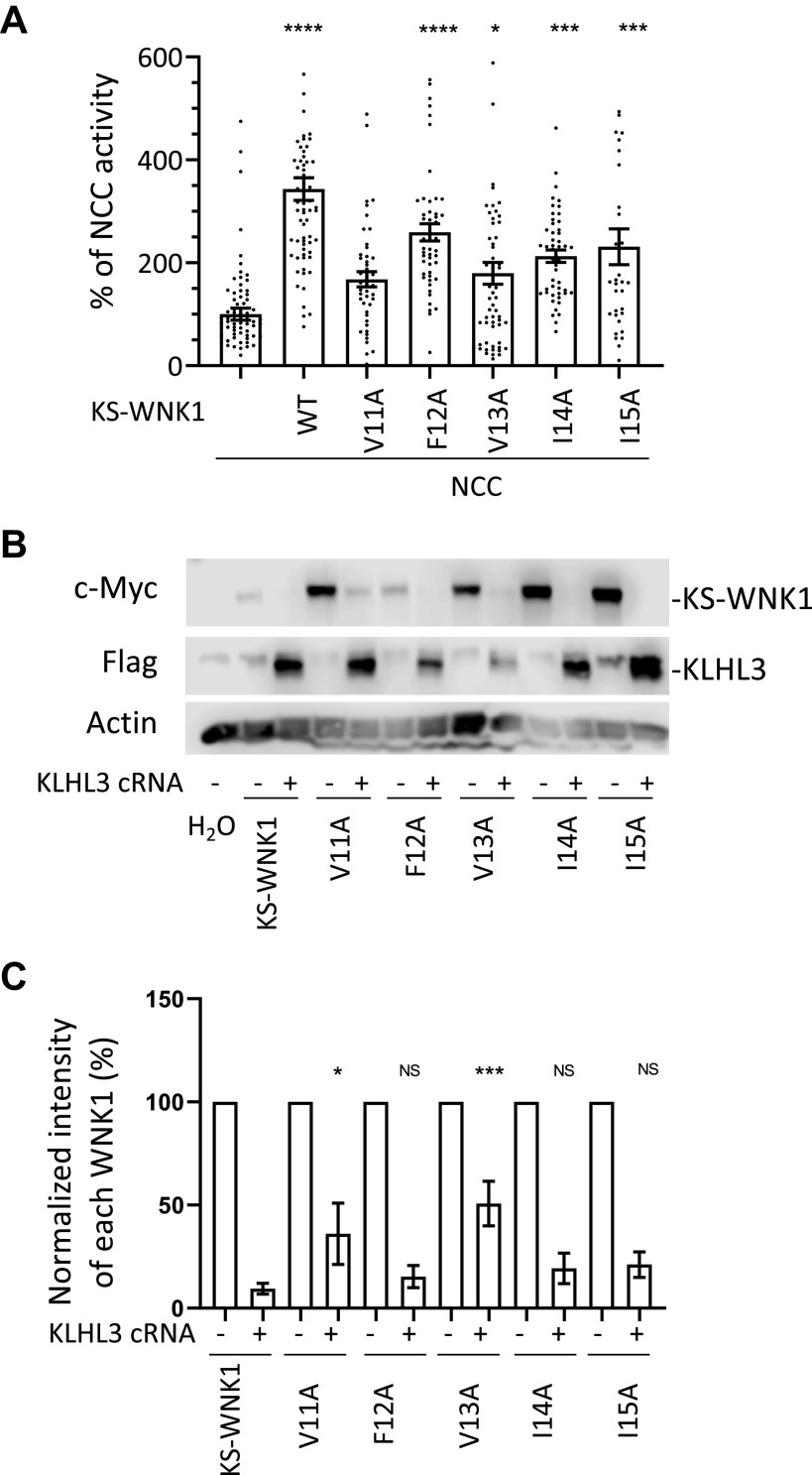
Mutation of valine 11 or valine 13 located in the hydrophobic motif of exon 4a impairs the ability of kidney-specific with no lysine kinase 1 (KS-WNK1) to activate NaCl cotransporter (NCC) and cullin-3-Kelch-like protein 3 (KLHL3) E3-induced degradation of KS-WNK1. *A*: NCC was coexpressed in oocytes with KS-WNK1 or with KS-WNK1 mutants containing one of the following single amino acid substitutions: V11A, F12A, V13A, I14A, or I15A. Uptake levels observed for NCC-expressing oocytes were normalized to 100% and compared with those observed for groups expressing KS-WNK1 mutants. All mutants except KS-WNK1-V11A were capable of activating NCC (*n* = 3−5 transport assays, *P < 0.05, ***P < 0.001, and ****P < 0.0001 vs. NCC; 16 points outside graphic limits). *B*: representative Western blots showing the expression of KS-WNK1 and of each single-residue mutant in the absence or presence of KLHL3. Compared with wild-type (WT) KS-WNK1, there was significantly less degradation of the V11A and V13A mutants in the presence of KLHL3. Other mutants degraded similarly to the WT. *C*: compiled results of densitometric analysis from at least five different experiments like that presented in *B*. Expression levels of KS-WNK1 and single-residue mutants in the absence of KLHL3 were normalized to 100%. Degradation of single-residue mutants were compared with WT KS-WNK1 in the presence of KLHL3 (*n* = 5−8 Western blots, *P < 0.05 and ***P < 0.001 vs. control with KLHL3).

To evaluate the role of individual residues within the hydrophobic cluster on KS-WNK1 activity and degradation, we generated the five single residue mutants and studied them in the oocyte system as performed for the other mutants. For the individual mutants, we decided to mutate each of the hydrophobic amino acid residues to alanine to explore whether the sole absence of the hydrophobic lateral chain is sufficient to produce these effects. We observed that the V11A mutant was unable to activate NCC and that this function was significantly reduced for the V13A mutant. For the remaining three mutants, this function was only slightly reduced (Fig. 8*A*). CUL3-KLHL3 E3-mediated degradation was impaired for the V11A and V13A mutants (Fig. 8, *B* and *C*), suggesting that these residues may participate in conferring the sensitivity to CUL3-KLHL3E3-mediated degradation.

### KS-WNK1 Protein Expression Is Upregulated in Kidneys of Mice Maintained on Low-K^+^ Diet

**Figure 9.**
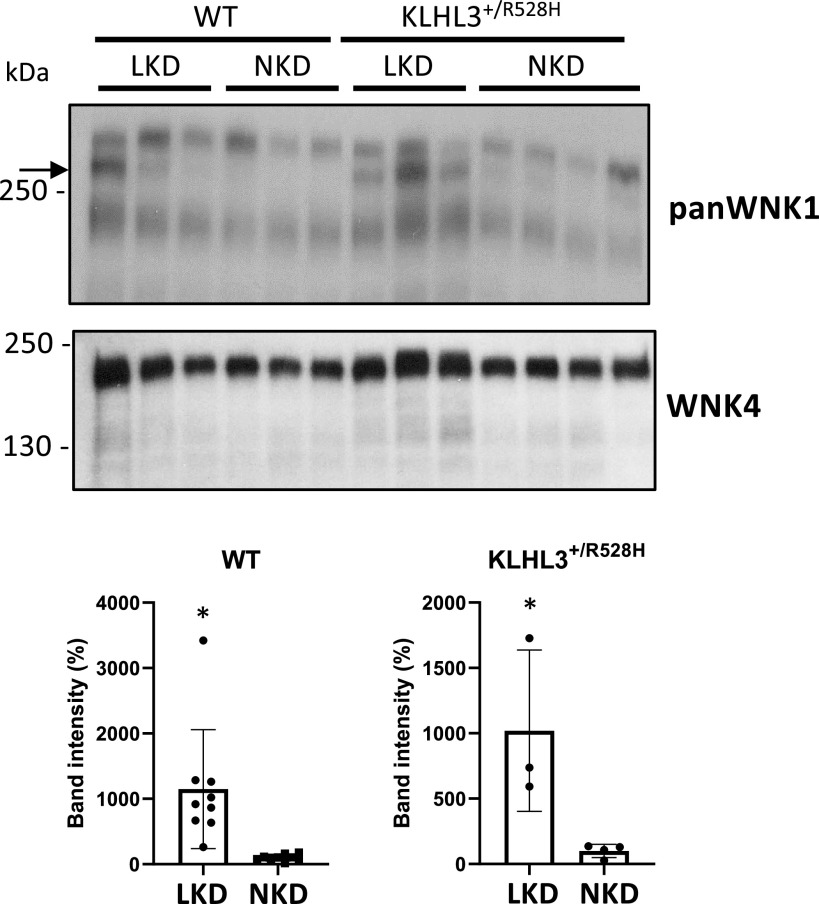
Renal kidney-specific with no lysine kinase 1 (KS-WNK1) expression is induced by a low-K^+^ diet (LKD). Total kidney lysates from wild-type (WT) and Kelch-like protein 3 (KLHL3)^+/R528H^ mice maintained on a normal-K^+^ diet (NKD) or LKD were analyzed by Western blot to assess the expression of KS-WNK1. Compared with WT mice, moderately higher expression was observed in KLHL3^+/R528H^ mice. In addition, a robust increase in KS-WNK1 expression was observed in WT mice but also in KLHL3^+/R528H^ mice, suggesting that KLHL3-targeted degradation was further affected under this condition. Results of quantitation of the band corresponding to KS-WNK1 are shown at the *bottom*. Band intensity values of mice on a NKD were normalized to 100%. A WNK4 blot is also shown. The expected increase in WNK4 expression was observed in KLHL3^+/R528H^ samples. Expression was further increased when mice were placed on a LKD, as previously reported (10). *n* = 9 for WT mice on the LKD and NKD, *n* = 3 for KLHL3^+/R528H^ mice on the NKD, and *n* = 4 for KLHL3^+/R528H^ mice on the LKD. *P < 0.05 versus the NKD. WNK1, with no lysine kinase 1.

Ishizawa et al. (23) recently showed that phosphorylation of KLHL3 in a residue located within the substrate-binding domain is upregulated in mice that are maintained on a low-K^+^ diet. This reduces CUL3-KLHL3 E3-targeted degradation of WNK4. Thus, to evaluate whether K^+^ restriction is a possible physiological stimuli for induction of KS-WNK1 expression, wild-type, and *KLHL3*^+/R528H^ mice were placed on low-K^+^ diet for 7 days and then euthanized for renal tissue collection. Kidney lysates were prepared and analyzed by Western blot analysis with pan-WNK1 and WNK4 antibodies (Fig. 9).

The band corresponding to KS-WNK1 was only barely observed in samples from *KLHL3*^+/R528H^ mice (Fig. 9), in contrast to what we previously observed with *KLHL3^R528H^*^/R528H^ mouse samples (Fig. 3). This suggests that the residual activity of the CUL3-KLHL3 E3 complex present in heterozygous mice is sufficient to almost entirely degrade KS-WNK1. A more robust band was observed in samples from wild-type and *KLHL3*^+/R528H^ mice maintained on the low-K^+^ diet, showing that indeed KS-WNK1 expression is induced under conditions of dietary K^+^ restriction. WNK4 upregulation was also observed, as previously reported (23).

## DISCUSSION

In the present study, we show that KS-WNK1 is more sensitive to CUL3-KLHL3 E3-mediated degradation than L-WNK1, and we began to explore the elements of their primary sequence that are responsible for this difference. Given that the known binding site for KLHL3 in WNK kinases is the acidic motif, the observation is puzzling as both proteins present the exact same binding site. Interestingly, we observed that removal of the unique sequence of KS-WNK1 encoded by exon 4a decreases its sensitivity to CUL3–KLHL3 E3-mediated degradation. This shows that targeting by KLHL3 of the COOH-terminal segment of WNK1 (comprising the sequence encoded from exon 5 until the end that includes the acidic motif) is not efficient unless this 30-amino acid residue segment is present. We also showed that this segment is critical for the ability of KS-WNK1 to activate NCC. Other works also support the key role of exon 4a for KS-WNK1 function. For instance, Argaiz et al. (12) showed that the ability of KS-WNK1 to activate NCC by promoting WNK4 phosphorylation is impaired by the removal of this segment, although KS-WNK1 binding to WNK4 was not affected, and Boyd-Shiwarski et al. (13) showed that exon 4a is necessary for the KS-WNK1-dependent formation of WNK bodies.

Interestingly, the analysis of the effects of mutation of certain conserved residues within exon 4a showed that some of these modifications alter KS-WNK1 ability to activate NCC, but not its targeting by KLHL3, whereas those that affect KLHL3 targeting also affect the ability to activate NCC. Thus, although the same segment is key to define the sensitivity to CUL3-KLHL3 E3 and the ability of KS-WNK1 to activate NCC, these properties can be dissociated, suggesting that they are not strictly dependent on one another.

Substitution of either one of two specific residues within the conserved hydrophobic motif in exon 4a was enough to prevent CUL3-KLHL3 E3-mediated degradation of KS-WNK1. The mechanisms underlying 4a segment’s role on KS-WNK1 activity and sensitivity to targeting by KLHL3 remain as open questions for future studies. Possible mechanisms are, for example, that this segment may be involved in establishing key interactions or that perhaps it could be a determinant to achieve a specific conformational state of the protein.

Our data suggest that KS-WNK1 is also highly sensitive to CUL3-KLHL3 E3-mediated degradation in vivo. Vidal-Petiot et al. (11) reported that KS-WNK1 mRNA levels are high in the kidney (higher than L-WNK1 mRNA levels). Despite this, we were unable to detect KS-WNK1 protein in kidney lysates from wild-type mice by Western blot analysis. However, robust KS-WNK1 protein expression was detected in lysates from *KLHL3^R528H^*^/R528H^ mice in which KLHL3-WNK binding is impaired. Thus, the low protein expression observed in wild-type mice may result from a high degradation rate of KS-WNK1. The physiological significance of this observation remains to be determined. Analogous biological phenomena have been described. For example, the ubiquitous transcription factor hypoxia-inducible factor-1α (HIF-1α) is normally undetectable at the protein level due to highly active proteasomal degradation that is dependent on the presence of O_2_ (24). HIF-1α is marked for degradation by a cullin–RING E3 ligase complex in which the von Hippel-Lindau (VHL) tumor suppressor protein acts as the substrate recognition element. VHL can only recognize HIF-1α when it is hydroxylated at two proline residues, and hydroxylation is dependent on the activity of prolyl-hydroxylases that are activated in the presence of O_2_. Thus, in conditions of hypoxia, hydroxylation is prevented and HIF-1α expression is rapidly induced, as well as expression of its target genes.

In the case of KS-WNK1, the physiological stimuli that can upregulate its expression remain to be elucidated. We show here, however, that one of these stimuli is dietary K^+^ restriction. Ishizawa et al. (23) have shown that KLHL3 phosphorylation in the substrate recognition domain is induced by low-K^+^ intake in mice. Thus, low-K^+^-induced phosphorylation of KLHL3 may underlie the observed upregulation of KS-WNK1 in mice on a low-K^+^ diet. This observation is in line with the fact that the formation of WNK bodies, which is induced by low-K^+^ intake, requires the presence of KS-WNK1, suggesting that indeed the DCT response to low-K^+^ intake involves KS-WNK1 upregulation due to KLHL3 inhibition by phosphorylation. This agrees with the observation that WNK bodies are present in KLHL3^R528H/R528H^ mice (Fig. 2) but not in mice with FHHt caused by mutations in WNK4 (14).

The physiological role of KS-WNK1 is currently a very controversial issue. However, knowledge of the conditions in which KS-WNK1 protein is expressed in the DCT may help guide experiments to uncover this physiological role. In the present work, we show that despite the high KS-WNK1 protein upregulation observed in FHHt mice due to a mutation in KLHL3, KS-WNK1 is not essential to develop the FHHt phenotype. That is, WNK4 overexpression appears to be sufficient to produce the disease, consistent with prior results (25).

Different lines of evidence obtained from a diversity of transgenic mouse models suggest that the WNK1 isoform expressed in the DCT is KS-WNK1 and that L-WNK1 is not normally present (1). Absence of WNK4 expression completely impairs the phosphorylation and activity of NCC (26), demonstrating that WNK4 absence cannot be compensated by L-WNK1 activity. Additionally, in KLHL3^R528H/R528H^ mice, expression of both L-WNK1 and WNK4 is increased but the FHHt phenotype is completely abrogated by elimination of WNK4, although L-WNK1 remains upregulated, strongly suggesting that L-WNK1 is not present in the DCT (25). Thomson et al. (14) have shown that, within the large WNK bodies observed in WNK4 knockout mice, no active WNK1 is present, as indicated by the lack of signal obtained with the pT-loop WNK antibody, supporting that the WNK1 product in the WNK bodies is KS-WNK1, not L-WNK1. Finally, it has been demonstrated that intronic *WNK1* deletions responsible for FHHt cause ectopic expression of L-WNK1 in the DCT (7), and this is the unique situation in which the absence of WNK4 does not result in NCC downregulation (18). Thus, given that L-WNK1 activity is less sensitive to inhibition by Cl^−^ than WNK4 (27, 28) and less sensitive to CUL3-KLHL3 E3-induced degradation, as shown by this work, it is likely that ectopic expression of L-WNK1 promotes higher levels of NCC activity at any given value of intracellular Cl^−^ concentration and activity level of the CUL3-KLHL3 E3 complex. In this regard, it is noteworthy that *WNK1* intronic deletions cause FHHt even in the absence of WNK4 (18), supporting that if L-WNK1 is expressed in the DCT, the presence of WNK4 would be irrelevant. In future work, it would be interesting to establish how low-K^+^ interferes with CUL3-KLHL3 E3-mediated degradation of KS-WNK1.

Finally, as mentioned in the introduction, Louis-Dit-Picard et al. (8) have shown that humans and mice with heterozygous mutations in the acidic motif of WNK1 display a mild FHH phenotype that is easily corrected with thiazide treatment in both species. They also showed that the SPAK/OSR1-NCC pathway is upregulated in *WNK1*^+/delE631^ mice. Thus, they proposed that NCC upregulation is the primary defect leading to the phenotypic alterations. Also, based on results from in vitro experiments, they proposed that the increased expression of KS-WNK1 in the DCT is largely responsible for NCC upregulation as KS-WNK1 abundance is preferentially affected by these mutations. The large WNK bodies observed in DCT cells of these mice also support this idea, as well as the in vitro and in vivo data presented in this work, showing that KS-WNK1 is very sensitive to CUL3-KLHL3 E3-induced degradation. However, as KS-WNK-1 activates NCC via WNK4, the effect of an increase in KS-WNK1 may be buffered by the amount of WNK4, causing only a mild activation of NCC. This activation is probably enough to cause slight hyperkalemia and volume retention but not enough to produce a rise in blood pressure.

In conclusion, our work shows, both in vivo and in vitro, that KS-WNK1 is highly sensitive to CUL3-KLHL3 E3-induced degradation, whereas L-WNK1 is much less sensitive. The high sensitivity of KS-WNK1 seems to be due to the presence of the unique segment encoded in exon 4a. This segment is also key for KS-WNK1 function and its ability to activate NCC. We propose that this exquisite sensibility of KS-WNK1 to targeting by KLHL3 may be relevant to achieve rapid induction of KS-WNK1 protein expression under certain conditions, one of which appears to be extracellular K^+^ depletion. The mechanisms by which exon 4a affects the activity and sensitivity to degradation of KS-WNK1 and the role that KS-WNK1 upregulation plays under conditions of dietary K^+^ deprivation remain to be explored.

## GRANTS

This work was supported by National Institute of Diabetes and Digestive and Kidney Diseases Grant DK51496 (to G.G.), Grants 87794, 101720, and A1-S-8290 from Conacyt Mexico (to M.C-C., M.C-B., and G.G., respectively), Grants IA203620 and RA202718 from PAPIIT UNAM and Loreal L'Oréal-UNESCO-AMC-CONALMEX “For Women in Science, 2019” (to M.C-C.), and Grant IN201519 from PAPIIT UNAM (to G.G.). M.O-F. was supported by a scholarship from Conacyt-Mexico and is a graduate student in the PECEM MD/PhD program of the Universidad Nacional Autónoma de México. G.G. is the guarantor of the study. D.R.A. is supported by Medical Research Council Grant MC_UU_12016/2.

## DISCLOSURES

No conflicts of interest, financial or otherwise, are declared by the authors.

## AUTHOR CONTRIBUTIONS

M.O-F., M.C-C., J.Z., E.R.A., L.R-V., N.A.B., M.C-B., D.R.A., and G.G. conceived and designed research; M.O-F., N.V., M.C-B., J.Z., O.A., E.R.A., F.L.d-T., A.M.d-O., A.S-N., and L.R-V. performed experiments; M.O-F., M.C-C., J.Z., O.A., E.R.A., F.L.d-T., A.M.d-O., A.S-N., L.R-V., N.A.B., N.V., M.C-B., D.R.A., and G.G. analyzed data; M.O-F., M.C-C., M.C-C., J.Z., E.R.A., D.R.A., G.G., A.M.d-O., and A.S.N. interpreted results of experiments; M.O-F., M.C-B., J.Z., N.V., and G.G. prepared figures; M.O-F., M.C-C., M.C-B., and G.G. drafted manuscript; M.O-F., M.C-C., M.C-C., J.Z., E.R.A., A.M.d-O., A.S-N., L.R.V., N.A.B., D.R.A., and G.G. edited and revised manuscript; M.O-F., M.C-C., E.R.A., F.L.d-T., A.M.d-O., J.Z., A.S-N., L.R.V., N.A.B., N.V., M.C-B., D.R.A., and G.G. approved final version of manuscript.
